# Association between Paraoxonases Gene Expression and Oxidative Stress in Hepatotoxicity Induced by CCl_4_

**DOI:** 10.1155/2014/893212

**Published:** 2014-11-17

**Authors:** Mohamed M. Hafez, Othman A. Al-Shabanah, Naif O. Al-Harbi, Mohamed M. Al-Harbi, Salim S. Al-Rejaie, Saad M. Alsurayea, Mohamed M. Sayed-Ahmed

**Affiliations:** ^1^Department of Pharmacology and Toxicology, College of Pharmacy, King Saud University, P.O. Box 2457, Riyadh 11451, Saudi Arabia; ^2^Riyadh Regional Laboratory and Blood Bank, Riyadh 11451, Saudi Arabia

## Abstract

*Objectives*. The purpose of the study is to evaluate the hepatoprotective effect of rutin in carbon tetrachloride- (CCl_4_-) induced liver injuries in rat model. * Methods*. Forty male Wistar albino rats were divided into four groups. Group I was the control group and received dimethyl sulphoxide (DMSO) and olive oil. Group II received rutin. Groups III was treated with CCl_4_. Group IV was administered rutin after 48 h of CCl_4_ treatment. Liver enzymes level, lipid profile, lipid peroxidation, and hydrogen peroxide were measured. The genes expression levels were monitored by real time RT-PCR and western blot techniques. *Results*. CCl_4_ group showed significant increase in alanine aminotransferase (ALT), aspartate aminotransferase (AST), thiobarbituric acid reactive substances (TBAR), hydrogen peroxide (H_2_O_2_), and lipid profile and a significant decrease in glutathione peroxidase (GPx), glutathione S transferase (GST), catalase (CAT), paraoxonase-1 (PON-1), paraoxonase-3 (PON-3), peroxisome proliferator activated receptor delta (PPAR-*δ*), and ATP-binding cassette transporter 1 (ABAC1) genes expression levels. Interestingly, rutin supplementation completely reversed the biochemical and gene expression levels induced by CCl_4_ to control values.* Conclusion*. CCl_4_ administration causes aberration of genes expression levels in oxidative stress pathway resulting in DNA damage and hepatotoxicity. Rutin causes hepatoprotective effect through enhancing the antioxidant genes.

## 1. Introduction

Liver is important in regulating metabolic functions and various physiological processes [1]. In addition, it is involved in detoxification of some drugs and xenobiotics which lead to an increase susceptible to the toxicity from these agents [2]. Oxidative stress is correlated with inflammation, cancer, and multiple organ toxicity [3]. Exposure to toxic chemicals can cause hepatocyte injuries through metabolic activation of reactive oxygen species (ROS), such as superoxide, hydroxy radicals, and H_2_O_2_ [4]. ROS can induce tissue injury via lipid peroxidation and enhance liver fibrosis by increasing collagen synthesis [5, 6]. Antioxidants enzymes act as free radical scavenging systems and provide first-line defense against ROS such as superoxide dismutase (SOD), CAT, GPx, and nutritional antioxidants [7]. Their role as protective enzymes is well known and is investigated extensively in in vivo models. CAT and GPx catalyze dismutation of the superoxide anion (O^2−^) to H_2_O_2_ and then to water thus providing protection against ROS.

The paraoxonase (PON) gene is a family that contains three members, PON1, PON2, and PON3 [8]. PON1 is synthesized primarily in liver and secreted into plasma and its natural physiological function is metabolism of toxic oxidized lipids of both low density lipoprotein (LDL) and HDL particles [9]. PON3 is predominantly expressed in liver and is associated with HDL. PON1 and PON3 share the lactonase activity and antioxidant property which participate in preventing LDL oxidation [10]. The enzymatic activities of PON1 and PON3 are different [11, 12]. Numerous studies are focused on the relationship between PON1/PON3 and the development of oxidative stress related diseases [13, 14]. PON2 has antioxidant properties and is more widely distributed [10, 15].

Monocyte chemoattractant protein-1 (MCP-1) plays an important role in inflammation by regulating the recruitment of monocytes into tissues and their subsequent differentiation to macrophages [16]. Therefore, its expression increased in chronic inflammatory diseases including liver injury [17, 18]. The MCP-1 overexpression in liver cirrhosis suggests that the protein involved in hepatic injury and fibrosis may downregulate the action of MCP-1 [16].

Carbon tetrachloride is a xenobiotic that is extensively used to study hepatotoxicity in animal models by initiating lipid peroxidation [19]. Bioactivation of phase I cytochrome P450 system induced by CCl_4_ can induce acute and chronic tissue injuries through formation of reactive metabolic trichloromethyl radicals (^•^CCl3) and peroxy trichloromethyl radicals (^•^OOCCl3). Trichloromethyl can react with sulfhydryl groups (glutathione and protein thiols) and antioxidant enzymes such as CAT and SOD. Trichloromethyl-free radicals overproduction enhances the membrane lipid peroxidation, finally leading to liver steatosis, fibrosis, or cirrhosis [20]. These free radicals can covalently bind to macromolecules such as proteins, lipids, and nucleic acids [19, 21].

Polyphenolic compounds such as flavonoids are markedly found in fruits, vegetables, and medicinal plants and play important role in detoxification of free radicals [22, 23]. Rutin, flavonoid glycosides, possesses different protective effects such as antitumor [24], anti-inflammatory [25], antimutagenic [26], and immunomodulating activities [27]; and hepatoprotection against CCl_4_-induced liver injuries [19]. The present study investigated the hepatoprotective effects of rutin on oxidative stress and inflammation via studying the PON1, PPAR-*δ*, and MCP-1 genes expression in hepatotoxic rat model induced by CCl_4_.

## 2. Materials and Methods

### 2.1. Kits and Chemicals

Carbon tetrachloride and rutin were purchased from Sigma Chemicals Co., USA. TBAR kit was purchased from Cayman Chemical Company (Cayman chemical, MI); H_2_O_2_ kit was purchased from BioVision Company (BioVision, Inc, CA, USA). Gene expression kit was purchased from Applied Biosystems (Applied Biosystems, CA, USA) and synthesized by Metabione Company.

### 2.2. Animal

Six-week-old male Wistar albino rats with average body weight 180–200 gm were obtained from the Animal Care Center, College of Pharmacy, King Saud University, Riyadh, Saudi Arabia. The animals were kept in ordinary cages under standard conditions of temperature (22 ± 1°C), humidity (50–55%), and a 12 h light/dark. They have free access to standard laboratory feed and water, according to the study protocol. All methods including euthanasia procedure were conducted in accordance with Guide for Care and Use of Laboratory Animals, Institute for Laboratory Animal Research, National Institute of Health (NIH publication no. 80–23; 1996) and it has been approved by Research Ethics Committee of Experimental Animal Care Center, College of Pharmacy, King Saud University, Riyadh, Saudi Arabia.

### 2.3. Experimental Design

The experimental design was performed according to Khan et al. [19]. To study the hepatoprotective effects of rutin, a total of 40 adult male Wistar albino rats were used and randomly divided into 4 groups of 10 animals each as follows.

- Group I (control group) received 3 mL/kg olive oil intraperitoneally (Monday and Thursday) and 3 mL/kg DMSO intragastrically using gavage twice a week for four weeks (Saturday and Wednesday).
- Group II (rutin group) was intragastrically treated with 70 mg/kg rutin in DMSO twice a week for four weeks (Saturday and Wednesday).
- Group III (CCl_4_ group) was intraperitoneally treated with 3 mL/kg CCl_4_ (30% in olive oil) twice a week (Monday and Thursday) for four weeks.
- Group IV (CCl_4_-rutin group) received 70 mg/kg rutin intragastrically, after 48 h of CCl_4_ treatment, twice a week for four weeks (Saturday and Wednesday).

After 24 hours of last treatment protocol, animals were killed by decapitation after exposure to ether, and blood samples were obtained and then serum was separated and kept at −80°C. The liver was immediately removed and washed by ice-cold saline solution. A part of liver was shock-frozen in liquid nitrogen and stored at −80°C until being used for gene expression analysis.

### 2.4. Bioassay Measurements

#### 2.4.1. Blood Chemistry

Serum levels of liver enzymes (AST, ALT), total cholesterol, HDL, and LDL were estimated by using commercially available diagnostic kits (Human, Wiesbaden, Germany).

#### 2.4.2. Serum Hydrogen Peroxide Concentration

Serum H_2_O_2_ concentration levels were measured by BioVision assay kit (BioVision, Inc., CA, USA) according to manufacturer's instructions. The principles based on the present of horse radish peroxidase, the OxiRed probe, react with H_2_O_2_ to produce a product with color that can be measured spectrophotometrically.

#### 2.4.3. Serum Thiobarbituric Acid Reactive Substances

Lipid peroxidation in serum sample was determined using TBARS assay kit (Cayman Chemical, MI) according to the manufacturer's instructions. Briefly, MDA standard curve was prepared by diluting 250 *μ*L MDA standard with 750 *μ*L water and then serial dilution that started from 0 *μ*m to 50 *μ*m was prepared. A mixture of 100 *μ*L of serum sample, standard and 100 *μ*L of SDS was first prepared. Four mL of color reagent was added to each mixture and boiled for an hour. After that, the reaction was stopped on ice for 10 min and centrifuged for 10 min at 1600 ×g; then 150 *μ*L of the supernatant was loaded in a 96-well plate and absorbance was read at 540 nm. TBARS concentration was calculated from MDA standard curve.

### 2.5. Detection of Gene Expression Level by Real Time PCR in Liver Tissues

#### 2.5.1. Total RNA Extraction

Total RNA was extracted from liver tissues by TRIzol method according to the standard protocol. Briefly, RNA was extracted by homogenization of liver tissues (Polytron; Kinematica, Lucerne, Switzerland) in TRIzol reagent (Invitrogen Life Technologies, UK) at maximum speed for 90–120 s. The homogenate was incubated for 5 min at room temperature. A 1 : 5 volume of chloroform was added, and the tube was vortexed and subjected to centrifugation at 12,000 g for 15 min. The aqueous phase was isolated, and the total RNA was precipitated by cold absolute ethanol. After centrifugation and washing, the total RNA was finally eluted in 20 *μ*L of RNase, DNase free water. The quantity was characterized using a UV spectrophotometer (NanoDrop 8000, Thermo Scientific, USA). The isolated RNA has a 260/280 ratio of 1.9–2.1.

#### 2.5.2. First-Strand cDNA Synthesis

First-strand cDNA was synthesized from 1 *μ*g total RNA in 20 *μ*L by reverse transcription using high capacity cDNA kit (Applied Biosystems, CA, USA) according to the manufacturer's instructions. Reverse transcription reaction consists of 2 *μ*L Oligo-dT (50 *μ*M), 2 *μ*L of 10x reverse transcriptase buffer, 0.8 *μ*L of deoxynucleoside triphosphate (25 mM), 1 *μ*L of RNase inhibitor (40 U/*μ*L), 1 *μ*L of MultiScribe Reverse Transcriptase (50 U/*μ*L), and RNase free dH_2_O, up to a final volume of 20 *μ*L. The cDNA was then stored at −20°C for the gene expression study.

Real time quantitative PCR was performed to detect the gene expression of GPx, CAT, GST, PON1, PON3, PPAR-*δ*, MCP-1, and ABAC1 in liver tissue using SYBR master mix (Applied Biosystems, CA, USA) and the reaction was performed on ABI PRISM 7500 Detection System (Applied Biosystems, USA). The program was set to run for one cycle at 95°C for 2 min, followed by 40 cycles at 95°C for 15 s and at 60°C for 1 min. The specificity of PCR amplification was confirmed by agarose gel electrophoresis and melting curve analysis. GAPDH was used as internal control for qRT-PCR. The primers were designed using Primer Express 3.0 software (Applied Biosystems, CA, USA) and listed in Table 1. Results of gene expression were analyzed using 2^−ΔΔCT^ method. Data were expressed as the mean fold changes ± SEM for three independent amplifications.

### 2.6. Western Blot Analysis

Liver tissues were washed with ice-cold PBS and the protein extracts were prepared using ice-cold cell lyses buffer supplemented with protease inhibitor cocktail (IBI SCIENTIFIC, Peosta, USA). Protein concentrations were measured using Bradford assay (Bio-Rad, CA, USA) according to the manufacturer's protocol. Proteins were separated on 10% sodium dodecyl sulfate-polyacrylamide (SDS-PAGE) gels and transferred to nitrocellulose membrane. The membrane was blocked with 5% skimmed milk in TBS-T (10 mM Tris-HCl, 150 mM NaCl, 0.25% Tween 20, pH 7.5) at room temperature for 2 h followed by incubation with 2 *μ*g/mL of primary antibody for GPx (sc-22145), CAT (sc-34285), GST (ab19256), PON-1 (sc-59646), PON-3 (sc-21156), PPAR-*δ* (ab8937), ABCA-1 (sc-58219), MCP-1 (sc-28879), and GAPDH (sc-32757) diluted in TBS and 5% skimmed milk overnight at 4°C. After washing with TBS-T buffer, the membrane was incubated with 1 *μ*g/mL of horseradish peroxidase (HRP) labeled secondary antibody diluted in TBS-T buffer for 2 h at room temperature, followed by three washes with TBS-T buffer. The detection was performed using chemiluminescence assay (Amersham, GE Healthcare, Life Sciences, UK). Membranes were exposed to X-ray film to observe the bands (Kodak, Rochester, NY). Protein bands in treated and untreated (control) groups were quantified using the Kodak Scientific ID software.

### 2.7. Statistical Analyses

Differences between obtained values (mean ± SEM, *n* = 10) were carried out by one-way analysis of variance followed by Tukey-Kramer multiple comparison using Graphpad Prism 5 software. The differences were considered statistically significant at *P* < 0.05.

## 3. Results

### 3.1. The Effect of CCl_4_, Rutin, and Their Combination on Lipid Profile and Liver Enzymes

ALT and AST serum levels were used as biochemical markers for early acute hepatotoxicity. The CCl_4_ group showed significant increase in AST (65 ± 1.2) and ALT (72 ± 2.2) compared to the control group (23.5 ± 1.8 and 24.2 ± 1.3, resp.) (*P* < 0.001). The rutin group showed no significant changes in liver enzymes compared to the control group. However, administration of rutin with CCl_4_ resulted in reversal of hepatic damage biomarker induced by CCl_4_ to its normal values.

In the CCl_4_ group serum cholesterol, triglycerides, and LDL levels were significantly increased by 42%, 21%, and 60%, respectively, while HDL concentration was decreased by 39% compared to the control group. Administration of rutin alone resulted in nonsignificant change in lipid profile compared to the control group. Interestingly, rutin supplementation in combination with CCl_4_ resulted in complete reversal of lipid profile levels induced by CCl_4_ to its normal values (Table 2).

### 3.2. The Effect of CCl_4_, Rutin, and Their Combination on TBAR and H_2_O_2_

The effect of CCl_4_, rutin, and their combination on lipid peroxidation biomarkers TBAR and H_2_O_2_, was shown in Table 2. In the CCl_4_ group, there was significant increase in TBAR by 167% (*P* < 0.0001) and in H_2_O_2_ by 308% (*P* < 0.01) compared to the control group. Administration of rutin alone showed nonsignificant changes in TBAR and H_2_O_2_ levels compared to the control group. However, CCl_4_ with rutin administration resulted in complete reversal of TBAR and H_2_O_2_ levels induced by CCl_4_ to their normal values. These changes in TBAR and H_2_O_2_ levels were significant compared to CCl_4_ group (*P* < 0.0001).

### 3.3. The Effect of CCl_4_, Rutin, and Their Combination on the Antioxidant Gene Expression

**Figure 1:**
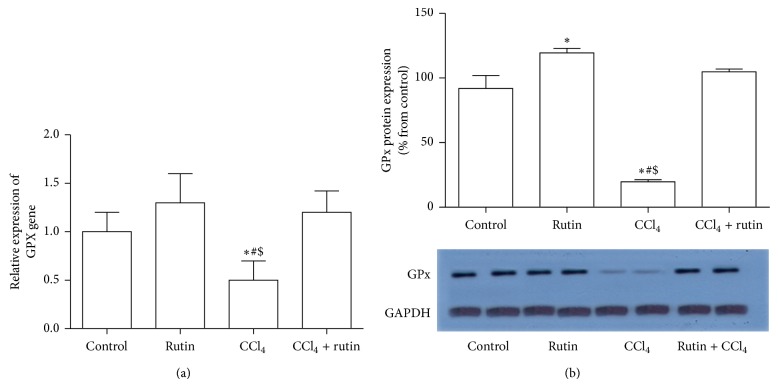
The effect of CCl_4_, rutin, and their combination on the mRNA (a) and protein (b) expression levels of glutathione peroxidase in rat liver. Data were presented as mean ± SEM (*n* = 10). ∗, #, and $ indicate significant change from control, rutin, and CCl_4_ plus rutin, respectively, at *P* < 0.05 using ANOVA followed by Tukey-Kramer as a post ANOVA test.

**Figure 2:**
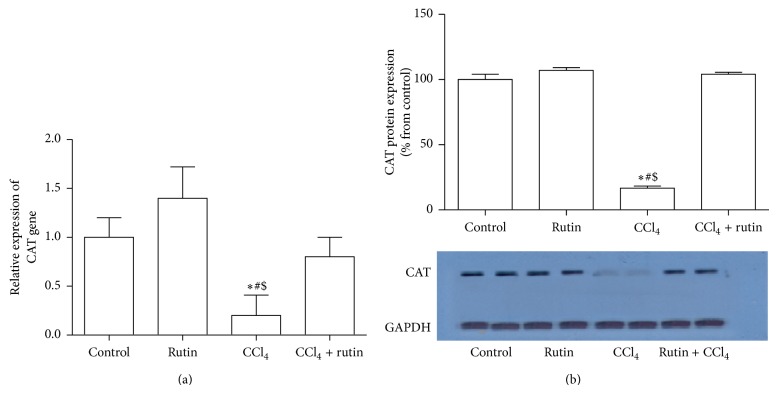
The effect of CCl_4_, rutin, and their combination on the mRNA (a) and protein (b) expression levels of catalase in rat liver. Data were presented as mean ± SEM (*n* = 10). ∗, #, and $ indicate significant change from control, rutin, and CCl_4_ plus rutin, respectively, at *P* < 0.05 using ANOVA followed by Tukey-Kramer as a post ANOVA test.

**Figure 3:**
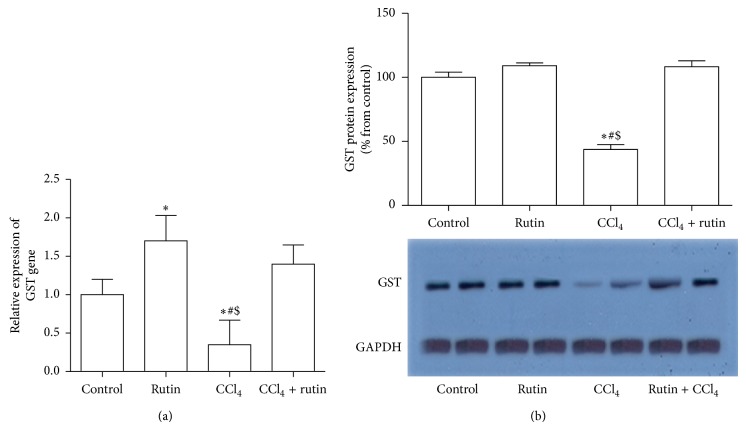
The effect of CCl_4_, rutin, and their combination on the mRNA (a) and protein (b) expression levels of glutathione S transferase in rat liver. Data were presented as mean ± SEM (*n* = 10). ∗, #, and $ indicate significant change from control, rutin, and CCl_4_ plus rutin, respectively, at *P* < 0.05 using ANOVA followed by Tukey-Kramer as a post ANOVA test.

To investigate the effect of CCl_4_, rutin, and their combination on the oxidative stress, the GPx, CAT, and GST genes expression levels were measured in liver tissues using real time RT-PCR and western blot analysis (Figures 1, 2, and 3). In the CCl_4_ group, GPx gene expression was significantly decreased in mRNA level by 2-fold (Figure 1(a)) and in protein level by 80% (Figure 1(b)); the CAT gene expression level was significantly decreased in mRNA by 5-fold (Figure 2(a)) and in protein level by 75% (Figure 2(b)) compared to the control group. Furthermore, in liver tissue, CCl_4_ significantly decreased the GST expression levels by 2.9-fold in mRNA (Figure 3(a)) and by 67% in protein level compared to the control group. Administration of rutin alone resulted in nonsignificant increase in GPx, CAT, and GST mRNA expression levels compared to the control group. Rutin supplementation in combination with CCl_4_ resulted in complete reversal of CCl_4_ aberrant effect on the antioxidant genes expression levels (mRNA and protein) to their normal values as in the control group. These reversal changes were observed as significant increase in GPx, CAT, and GST mRNA expression by 2.4-, 4-, and 4.4-fold and protein level by 85%, 88%, and 64% expression levels, respectively, compared to CCl_4_ group.

### 3.4. The Effect of CCl_4_, Rutin, and Their Combination on PON1 Gene Expression

**Figure 4:**
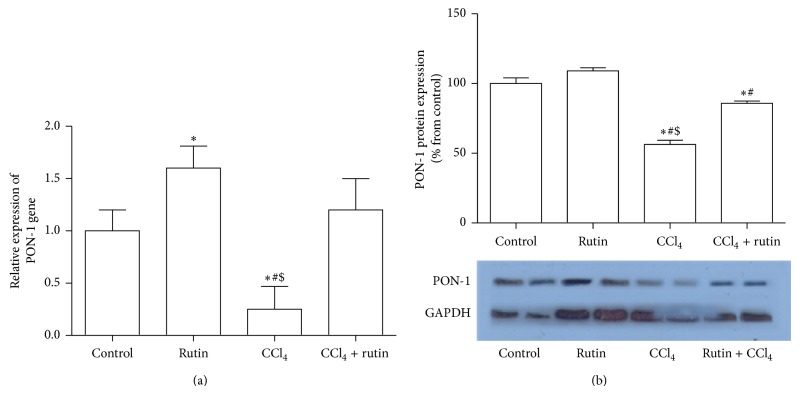
The effect of CCl_4_, rutin, and their combination on the PON-1 mRNA (a) and protein (b) expression levels in rat liver. Data were presented as mean ± SEM (*n* = 10). ∗, #, and $ indicate significant change from control, rutin, and CCl_4_ plus rutin, respectively, at *P* < 0.05 using ANOVA followed by Tukey-Kramer as a post ANOVA test.

Figure 4 showed the effect of CCl_4_, rutin, and their combination on the PON-1 gene expression level in liver tissue. In the CCl_4_ group significant reduction in the mRNA by 4-fold and protein by 50% expression level of PON-1 gene was observed (*P* < 0.02) (Figures 4(a) and 4(b)) compared to the control group. The supplementation of rutin in combination with CCl_4_ showed significant increase in the gene expression level by 4.8-fold (*P* < 0.02) compared to the CCl_4_ group, whereas the protein level in CCl_4_-rutin group was significantly decreased compared to both control and rutin groups. In the rutin group, there was a significant increase in the PON-1 gene expression level compared to the control group (*P* < 0.05).

### 3.5. The Effect of CCl_4_, Rutin, and Their Combination on the PON-3 Gene Expression Level

**Figure 5:**
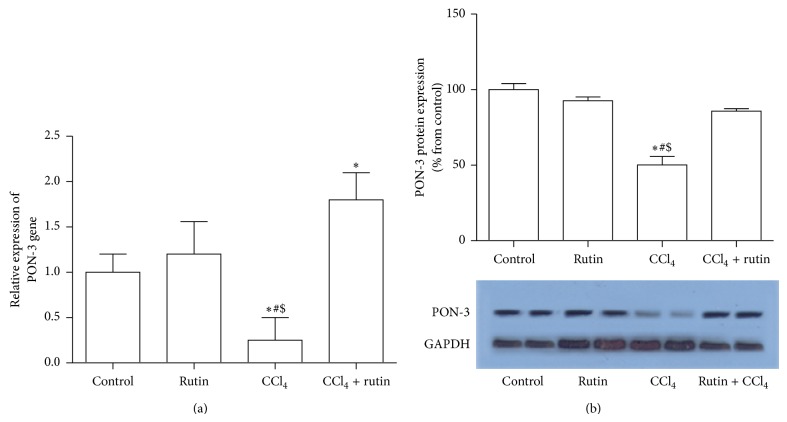
The effect of CCl_4_, rutin, and their combination on the PON-3 mRNA (a) and protein (b) expression levels in rat liver. Data were presented as mean ± SEM (*n* = 10). ∗, #, and $ indicate significant change from control, rutin, and CCl_4_ plus rutin, respectively, at *P* < 0.05 using ANOVA followed by Tukey-Kramer as a post ANOVA test.

The effect of CCl_4_, rutin, and their combination on PON-3 expression level was shown in Figure 5. The administration of CCl_4_ alone resulted in a decrease in the expression level of PON-3 mRNA by 4-fold (Figure 5(a)) and protein by 52% (Figure 5(b)) (*P* < 0.03) compared to the control group. The administration of rutin in combination with CCl_4_ showed a significant increase in the mRNA expression level by 1.8- (*P* < 0.03) and 7.2- (*P* < 0.001) fold compared to the control and CCl_4_ groups, respectively.

### 3.6. The Effect of CCl_4_, Rutin, and Their Combination on ABCA1 Gene Expression

**Figure 6:**
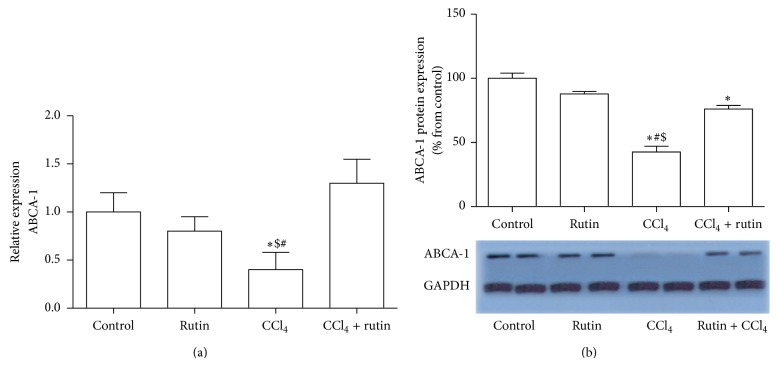
The effect of CCl_4_, rutin, and their combination on the mRNA (a) and protein (b) expression levels of ABCA1 gene in rat liver. Data were presented as mean ± SEM (*n* = 10). ∗, #, and $ indicate significant change from control, rutin, and CCl_4_ plus rutin, respectively, at *P* < 0.05 using ANOVA followed by Tukey-Kramer as a post ANOVA test.

Figure 6 showed the effect of CCl_4_, rutin, and their combination on the expression level of ABCA1 gene. In the CCl_4_ group, ABCA1 was significantly decreased in mRNA by 2.5-fold expression (Figure 6(a)) and in protein level by 58% (Figure 6(b)) compared to the control group (*P* < 0.038). The administration of rutin in combination with CCl_4_ resulted in significant increase in the mRNA by 3.25-fold and protein expression levels by 25% of ABCA1 gene compared to CCl_4_ group (*P* < 0.009). In CCl_4_ with rutin supplementation, the increase in ABCA1 mRNA was insignificant but the protein level was significantly compared to the control group.

### 3.7. The Effect of CCl_4_, Rutin, and Their Combination on PPAR-*δ* Gene Expression

**Figure 7:**
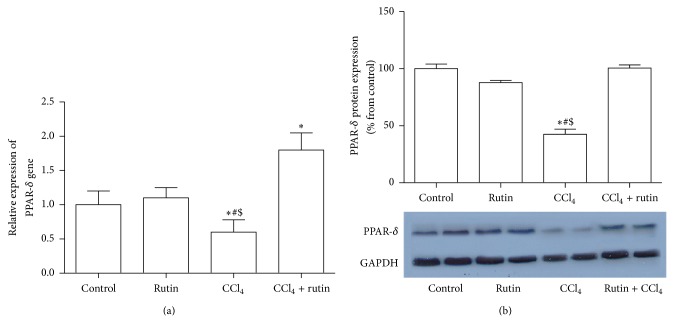
The effect of CCl_4_, rutin, and their combination on the mRNA (a) and protein (b) expression levels of PPAR-*δ* in rat liver. Data were presented as mean ± SEM (*n* = 10). ∗, #, and $ indicate significant change from control, rutin, and CCl_4_ plus rutin, respectively, at *P* < 0.05 using ANOVA followed by Tukey-Kramer as a post ANOVA test.

Figure 7 showed the effect of CCl_4_, rutin, and their combination on PPAR-*δ* gene expression in liver tissues. In the CCl_4_ group, the PPAR-*δ* gene expression was significantly decreased in mRNA by 1.6-fold (Figure 7(a)) and protein by 56% (Figure 7(b)) compared to the control group. The administration of rutin in combination with CCl_4_ showed significant increase in the PPAR-*δ* expression level mRNA 3-fold (*P* < 0.002) and protein by 58% compared to CCl_4_ groups. The increase in mRNA PPAR- *δ* in CCl_4_-rutin group was significantly compared to the control group (*P* < 0.03).

### 3.8. The Effect of CCl_4_, Rutin, and Their Combination on MCP-1 Gene Expression

**Figure 8:**
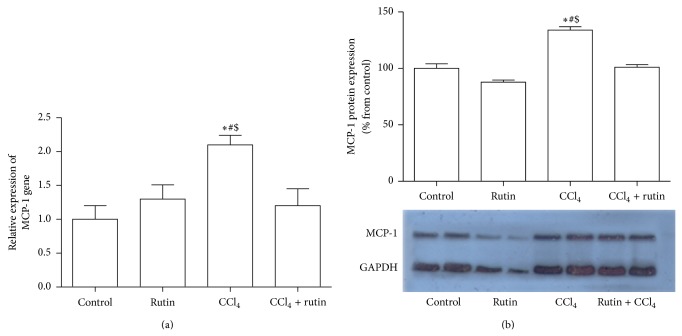
The effect of CCl_4_, rutin, and their combination on the mRNA (a) and protein (b) MCP-1 expression levels in rat liver. Data were presented as mean ± SEM (*n* = 10). ∗, #, and $ indicate significant change from control, rutin, and CCl_4_ plus rutin, respectively, at *P* < 0.05 using ANOVA followed by Tukey-Kramer as a post ANOVA test.

The effect of CCl_4_, rutin, and their combination on MCP-1 gene expression in liver tissues was shown in Figure 8. In the CCl_4_ group, the expression level of MCP-1 was significantly increased in mRNA by 2.1-fold and protein by 34% compared to the control group. In the CCl_4_-rutin group a significant decrease was observed in the MCP-1 mRNA and protein expression. This decrease in the expression level was statistically significant compared to CCl_4_ group. In the rutin group, the MCP-1 mRNA expression level was insignificantly increased compared to the control group (*P* < 0.3).

## 4. Discussion

Liver diseases caused by viral infection or other hepatotoxic agent are highly associated with severe damage [28, 29]. Hepatic inflammation is considered as early stage of fibrosis, which can progress to extensive fibrosis and cirrhosis. Carbon tetrachloride is widely used to investigate liver injury associated with oxidative stress and free radicals. Reactive oxygen species induced by CCl_4_ not only cause direct tissue damage but also initiate inflammation [30]. Chemoprevention and dietary modification are effective against oxidative stress and are now the focus of several researches [31]. Rutin had hepatoprotective effect against agents-induced liver injuries.

The present study showed that CCl_4_ significantly increased the ALT and AST serum levels. The increase in the liver enzymes resulted in acute hepatocyte injuries caused by CCl_4_ [32]. In the current study, rutin supplementation in combination with CCl_4_ significantly restored the AST and ALT levels to the normal values. Therefore, rutin may have the ability to protect liver from CCl_4_-induced injury. Similarly, previous study reported the protective effect of flavonoid compounds against CCl_4_-induced hepatotoxicity [19].

The CCl_4_-induced oxidative stress causes DNA mutation and increases fibroblastic activity leading to liver cirrhosis and carcinoma. In the present study, the aberrations in lipid profile induced by CCl_4_ were restored to normal values with rutin treatment. Lipid alteration is a causal factor for oxidative stress that resulted from increase in ROS production and reduction in antioxidant enzymes [33]. Lipid peroxidation and ROS impaired the respiratory chain in hepatocyte via oxidative damage of mitochondrial DNA. The rutin protective effect is due to the ability to chelate metal ions and minerals hence decreasing oxidative stress and lowering lipid profile [34]. This hypothesis is in agreement with another study that found hepatoprotective effects of some plant bioactive compounds against CCl_4_-induced hepatic injury in rats [35]. Lipid peroxidation is characterized by imbalance between oxidant-antioxidant and ROS induced by CCl_4_.

Glutathione in liver provides the first line of defense by scavenging ROS. The decrease in glutathione concentration may be due to NADPH reduction or GSH utilization in exclusion of peroxides [36]. In the current study, lipid peroxidation was increased in CCl_4_ group by increasing TBAR serum level. This will cause H_2_O_2_ elevation that could further stimulate lipid peroxidation. Similarly, another study showed that CCl_4_ is an independent risk factor for increasing lipid peroxidation and decreases activity of antioxidant enzymes which lead to DNA damage [19]. In the current study, administration of rutin with CCl_4_ markedly reduced the DNA damage via reduction in oxidative stress, which agrees with previous study [37].

Cell damage, induced by oxidative stress, is attenuated by antioxidant enzyme. When the imbalance between ROS production and antioxidant defense is lost, oxidative stress occurred through a serious of events which deregulates the cellular functions leading to various pathological conditions [38]. Glutathione peroxidase, glutathione S transferase, and catalase enzymes are superoxide ion and H_2_O_2_ scavengers that protect cells from oxidative damage [39]. Catalase removes the peroxide produced by superoxide dismutase and catalyses the breakdown of H_2_O_2_ to water and oxygen. The current study showed significant decrease in GPx, GST, and CAT gene expression levels in liver tissues in association with increase in serum TBAR and H_2_O_2_ in CCl_4_ group and is in agreement with another study [39]. Lipid oxidation product (TBAR) and antioxidants genes GSP, CAT, and GST aberrations induced by CCl_4_ were restored to normal levels by rutin treatment.

The PON1 and PON3 are efficient in reducing LDL oxidation [40]. The decreased expression and activity of PON1 have been associated with chronic liver disease [41]. The present data revealed significant decrease in PON1 and PON3 expression levels in CCl_4_-group in liver tissues. PON1 and PON3 are mainly synthesized and expressed in liver [42, 43]. In the present study, the decrease in PON1 and PON3 expression may be due to the hepatic dysfunction induced by CCl_4_ toxicity. PON1 protein, an antioxidant, is localized on HDL surface and stimulates the macrophage cholesterol efflux. The decrease in PON1 expression and activity could be a consequence of altered synthesis and/or secretion of HDL. This alteration in chronic liver disease is associated with decrease in hepatic lecithin cholesterol acyl transferase activity [44].

Carbon tetrachloride metabolism produces high reactive free radicals that react with sulfhydryl groups, such as glutathione and protein thiols [45]. Antioxidant activity of PON1 is associated with its –SH groups; therefore, the reduction in PON1 antioxidant activity might be due to alteration in nature and number of free thiol groups in its molecule [46]. These data support the idea that administration of CCl_4_ induces oxidative stress by increasing lipid peroxidation which in turn decreases PON1 expression.

The hepatoprotective effect of PON3 was closely interrelated to its lactonase activities and antioxidant capabilities. PON3 can reduce oxidative products via preventing oxidation and suppressing the propagation of oxidation by destroying lipid hydroperoxides in oxidized LDL [47, 48]. In the current study, PON1 and PON3 genes were significantly increased in rutin supplementation with CCl_4_ treated rats. Data from this study suggested that PON1 and PON3 might have protective effect on hepatotoxicity induced by CCl_4_ via hydrolyzing specific oxidized lipids.

The MCP-1 production by oxidized lipids and lipoproteins is important in inflammation [49]. PON1 inhibited the production of MCP-1 induced by oxidized LDL [50]. PON1 and MCP-1 are collaborated in regulating inflammatory processes. Increased MCP-1 concentration and decreased PON1 activity are often observed in conditions involving oxidative stress [51]. Aldehyde, the end-product of lipid peroxidation, acts as a regulator to MCP-1 expression in liver [18]. In the present study, a significant increase in MCP-1 gene expression was associated with hepatotoxicity. Similarly, high MCP-1 gene expression level was reported in patients with chronic hepatitis or liver cirrhosis [52, 53] and is correlated with monocyte infiltration in liver. In liver, MCP-1 expression may be initiated or upregulated by circulating lipopolysaccharide or other members of cytokines such as tumor necrosis factor alpha and interleukin-1 [54]. MCP-1 upregulation by oxidizing lipids and lipoproteins is an important factor in initial stages of inflammation [55]. PON1 may act as a barrier against hepatic oxidative stress. This barrier is overcome by exposure to CCl_4_ leading to increase in MCP-1 with severe proinflammatory reaction. PON1 attenuates MCP-1 production induced by oxidized LDL when incubated with epithelial cells [56]. The mechanisms for PON1 and MCP-1 regulation are still unknown.

Peroxisome proliferator-activated receptors (PPARs) are group of nuclear receptor proteins that regulate expression of many genes [57]. PPARs play important roles in metabolism, differentiation, and cancer [58, 59]. PPARs may be involved in regulating and coordinating PON1 and MCP-1 genes expression; PPARs may upregulate PON1 expression in a variety of clinical and experimental situations. Recent evidence indicates that PPAR downregulates MCP-1 expression [50, 60]. In the present study, the PPAR-*δ* expression level was significantly decreased in CCl_4_ group compared to the control group. Similarly, another study found that the expression level of PPAR*δ* (protein and mRNA) was decreased following CCl_4_ administration [61]. PPAR*δ* increases HDL synthesis through activation of ABCA1 gene (the cellular cholesterol exporter) [62]. In the present study, the decrease in ABCA1 gene expression level in CCl_4_ group may explain the highest level of cholesterol in the same group. Similarly, others found that ABCA1 can mediate the phospholipids and cholesterol efflux and can eliminate HDL biosynthesis in liver [63, 64]. The current study suggested that the decrease in PPAR-*δ* gene expression following CCl_4_ administration may be associated with decrease in HDL synthesis. PPAR-*δ* can upregulate the expression of several antioxidant genes including SOD, CAT, and thioredoxin. The PPAR-*δ* suppression is proapoptotic mechanism to eliminate damaged hepatocyte induced by CCl_4_ [65].

## 5. Conclusion

In conclusion, the present study suggests that the effect of rutin on CCl_4_-induced hepatotoxicity was mostly by reducing oxidative stress and inflammation in liver. Rutin may be used as an antioxidant via decreasing hepatic stress induced by toxic agents. Also, the present study suggests that PON1, PON3, and PPAR-*δ* had protective roles against liver disease.

## Figures and Tables

**Table 1:** The primers used for real time polymerase chain reaction.

Gene	Forward primer	Reverse primer
PON-1	TGAGAGCTTCTATGCCACAAATG	CCATGACAGGCCCAAGTACA
PON-3	CATCCAGGATCCTTTGTCAGATAA	CACGGTGCTGCCCTGAAG
CAT	CGACCGAGGGATTCCAGATG	ATCCGGGTCTTCCTGTGCAA
GPx	CGGTTTCCCGTGCAATCAGT	ACACCGGGGACCAAATGATG
GST	GCC TTC TAC CCG AA G ACA CCT T	GTC AGC CTG TTC CCT ACA
MCP-1	TCGCTTCTGACACCATGCA	TGCTACAGGCAGCAAATGTGA
ABCA1	CCCGGCGGAGTAGAAAGG	AGGGCGATGCAAACAAAGAC
PPAR-*δ*	GCCAAGAACATCCCCAACTTC	GCAAAGATGGCCTCATGCA
GAPDH	AACTCCCATTCCTCCACCTT	GAGGGCCTCTCTCTTGCTCT

**Table 2:** The effect of CCl_4_, rutin, and their combination on lipid profile, TBAR, and H_2_O_2_.

Group	Cholesterol (mg/dL)	Triglycerides (mg/dL)	HDL (mg/dL)	LDL (mg/dL)	TBAR (nmol/mL)	H_2_O_2_ (*µ*M)
Control	50 ± 0.33	57.82 ± 1.9	33.4 ± 0.3	38 ± 0.95	18 ± 2.2	1.3 ± 1.0
Rutin	56 ± 0.8^#^	52 ± 0.45^#^	36.6 ± 0.53^#^	35 ± 0.32^#^	22 ± 1.8^#^	1.2 ± 1.1^#^
CCl_4_	71.2 ± 0.68^*^	69 ± 0.58^*^	20.8 ± 0.8^*^	54 ± 0.8^*^	48 ± 3.2^*^	5.3 ± 1.2^*^
CCl_4_ + rutin	52 ± 0.52^#^	48 ± 0.90^#^	38 ± 0.78^#^	32 ± 0.9^#^	25 ± 2.1^∗#^	1.5 ± 1.5^#^

Mean ± SE (*n* = 10); HDL: high density lipoprotein; LDL: low density lipoprotein.

^*^indicates significance from the control group.

^#^indicates significance from the CCl_4_ group.
